# ABO Incompatible Kidney Transplantation Without B-cell Depletion is Associated With Increased Early Acute Rejection: A Single-Center Australian Experience

**DOI:** 10.3389/ti.2023.11567

**Published:** 2023-09-20

**Authors:** Jonathan M. Bleasel, Susan S. Wan, Steven J. Chadban, Tracey Ying, David M. Gracey, Leyla J. Aouad, Qian-Ao Chen, Mike Utsiwegota, Jane Mawson, Kate R. Wyburn

**Affiliations:** ^1^ Department of Renal Medicine, Royal Prince Alfred Hospital, Sydney, NSW, Australia; ^2^ Kidney Node, Charles Perkins Centre, University of Sydney, Sydney, NSW, Australia; ^3^ Central Clinical School, Faculty of Medicine and Health, The University of Sydney, Sydney, NSW, Australia

**Keywords:** ABO incompatible, kidney transplantation, rituximab, immunosuppression, rejection

## Abstract

We performed a single-center retrospective cohort study of 66 consecutive ABO incompatible kidney transplants (ABOiKT) performed without B-cell depleting therapy. Outcomes were compared to an earlier era performed with rituximab (*n* = 18) and a contemporaneous cohort of ABO compatible live donor transplants (ABOcKT). Acute rejection within 3 months of transplant was significantly more common after rituximab-free ABOiKT compared to ABOiKT with rituximab (OR 8.8, *p* = 0.04) and ABOcKT (OR 2.9, *p* = 0.005) in adjusted analyses. Six recipients of rituximab-free ABOiKT experienced refractory antibody mediated rejection requiring splenectomy, and a further two incurred early graft loss with no such episodes amongst ABOiKT with rituximab or ABOcKT cohorts. Patient and graft survival were similar between groups over a median follow-up of 3.1 years. This observational evidence lends strong support to the continued inclusion of rituximab in desensitization protocols for ABOiKT.

## Graphical Abstract

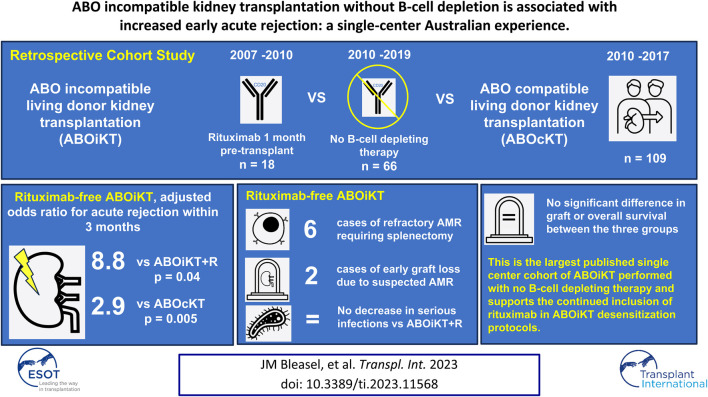

## Introduction

Kidney transplant offers the best survival and quality of life for most patients with end stage kidney disease [1, 2]. Limited availability of living donors and long waiting times for deceased donor allocation leave many transplant candidates to accrue significant morbidity and healthcare expenditure on dialysis [3, 4]. Since the pioneering case series using extracorporeal antibody removal and splenectomy [5], kidney transplantation between ABO incompatible individuals (ABOiKT) has developed as a viable strategy to increase the living donor pool. The anti-CD20 monoclonal antibody rituximab has now replaced splenectomy as pre-transplant B-cell depleting therapy in almost all ABOiKT programs, with excellent outcomes in terms of rejection and graft survival reported by individual centers [6–8]. However, increased rates of infection and death from infection have been observed in ABOiKT recipients compared to their ABO compatible counterparts, raising concerns about the degree of immunosuppression required for the procedure [9–12]. Our center initiated an ABOiKT program in 2007 employing pretransplant rituximab and immunoadsorption. Three years later rituximab was excluded from the desensitization protocol due to concerns about infection risk and following reports of successful ABOiKT with no B-cell depleting therapy at other centers [13, 14]. Here we evaluated outcomes of rituximab-free ABOiKT through comparison with the earlier era of our ABOiKT program where rituximab use was universal (ABOiKT + R), and a contemporaneous cohort of living donor ABO compatible transplants (ABOcKT).

## Materials and Methods

### Study Design and Setting

The Royal Prince Alfred Hospital is a tertiary referral center in Sydney, Australia with a kidney transplant unit servicing a large metropolitan district as well as a number of rural centers. We conducted a retrospective cohort study of ABOiKT performed from the inception of the program on 1 July 2007 until 1 June 2019. A near contemporaneous comparator cohort of ABOcKT (July 2010 and April 2017) with prospectively collected outcome data was adopted from a previously published study of donor specific antibodies in kidney transplantation [15].

This study received ethical approval from the Sydney Local Health District Human Research Ethics Committee (reference X17-0083 and LNR/17/RPAH/124).

### Desensitization Protocol for ABO Incompatible Kidney Transplants

Prospective ABOiKT recipients between July 2007 and July 2010 (*n* = 18) received rituximab 375 mg/m^2^ 1 month pre-operatively. After this era, rituximab was omitted from the desensitization protocol and 66 further ABOiKT were performed with no other changes to immunosuppression practices. ABOiKT recipients commenced mycophenolate mofetil 1,000 mg twice daily 14 days prior to transplantation. Anti-A or B antibody removal was achieved by immunoadsorption (Glycosorb A/B®, Glycorex Transplantation AB, Sweden). Immunoadsorption sessions were scheduled according to baseline blood group antibody titer to achieve a preoperative titer of 1:8 or less. A single dose of 500 mg/kg intravenous immunoglobulin (IVIg) was administered on the day prior to transplant. Post-operative immunoadsorption sessions were performed only in cases of antibody titer rebound to greater than 1:8 or suspected anti-blood group antibody mediated rejection.

### Induction and Maintenance Immunosuppression

Routine induction immunosuppression was the same for ABOiKT in both eras and ABOcKT, consisting of two doses of intravenous (IV) basiliximab 20 mg (day zero and day 4 post-operatively) and methylprednisolone 500 mg IV on day zero and 250 mg IV on day 1. Highly sensitized recipients considered to be at significant risk of rejection received anti-thymocyte globulin instead of basiliximab as induction therapy. Standard maintenance immunosuppression in all recipients was mycophenolate mofetil 1000 mg twice daily, a calcineurin inhibitor (tacrolimus or ciclosporin) and prednisolone starting at 30 mg daily and weaning to 10 mg daily by 8 weeks post-transplant.

A protocolized kidney transplant biopsy was performed on day 10 after ABOiKT if no indication biopsy had been performed prior, and another was performed at week 12. ABOcKT recipients had a protocolized biopsy at week 12 only.

All transplant recipients received *pneumocystis jirovecii* prophylaxis with trimethoprim/sulfamethoxazole or an alternative agent indefinitely while immunosuppressed. Cytomegalovirus prophylaxis with oral valganciclovir was employed for 3–6 months depending on risk of cytomegalovirus reactivation.

### Anti-blood Group Antibody Measurements and Alloantibody Detection

Anti-A and B antibody titers in ABOiKT recipients were measured by column agglutination technology using DG Gel® cards and reagent red blood cells (Grifols, Melbourne, Australia). Complement dependent cytotoxic (CDC) cross matching, flow cytometric cross matching and solid-phase Luminex assay for anti-HLA donor specific antibodies (One Lambda LABScreen Single Antigen class I and II; BMT, Mehrbusch, Germany) were performed by the Australian Red Cross New South Wales transplantation and immunogenetics lab in accordance with international guidelines [16]. The threshold for reporting anti-HLA antibody positivity was mean fluorescence intensity (MFI) >500.

Prospective ABOiKT or ABOcKT recipients with high level donor specific antibodies (DSAs) (MFI >3,000) or positive CDC T-cell cross match were directed toward alternative transplant pathways wherever possible. Low or intermediate strength DSAs with negative cross match were accepted and pre-transplant therapeutic plasma exchange was employed in selected cases.

### Clinical Data Collection

Data were extracted from the clinical record and managed using REDCap electronic data capture tools hosted at Sydney Local Health District [17, 18]. Delayed graft function (DGF) was defined as requirement for dialysis within 7 days of transplant. Graft failure was defined as need to return to dialysis permanently, re-transplantation or estimated glomerular filtration rate <15 mL/min/1.73 m^2^ sustained for at least 6 weeks. Rejection was defined according to Banff criteria [19]; only treated episodes of biopsy proven rejection were recorded for this analysis. Early rejection was defined as occurring within 3 months of transplant.

### Statistical Analysis

Open-source statistical software R (http://r-project.org) was used for all statistical analysis. Between group comparisons were performed using Student’s t-test or Wilcoxon Rank Sum test for parametric and nonparametric continuous data, respectively, and Fisher’s exact test for categorical data. Acute rejection was analyzed using logistic regression. Base models included all covariates with a univariable *p*-value ≤0.25, and a backward elimination strategy was employed to determine the final model. Death censored graft survival and overall survival were calculated using Kaplan-Meier survival tables and compared between groups using cox proportional hazards models.

## Results

### Patient Characteristics

Sixty-six ABOiKT were performed without B-cell depleting therapy between July 2010 and June 2019. The comparator groups comprise 18 ABOiKT performed with pre-transplant rituximab between July 2007 and July 2010 and 109 consecutive ABOcKT transplants. Median follow-up for the whole cohort was 3.1 years (IQR 1.3–5.0 years).

Baseline characteristics of the three groups are presented in Table 1. Recipient age, sex, race, and cause of ESKD were similarly distributed between the three groups. Donor age was older in the rituximab-free ABOiKT group (52 years) compared to the earlier ABOiKT era (46 years) and ABOcKT (49 years).

**TABLE 1:** Baseline characteristics of study participants. All numbers refer to frequency and percentage unless otherwise described.

	ABOiKT		
	Rituximab-free *n* = 66	Rituximab *n* = 18	ABOcKT *n* = 109
Age in years (mean, SD)	47.9 (13.9)	43.1 (12.9)	45.5 (14.9)
Male recipient	47 (71.2%)	13 (72.2%)	73 (67.0%)
Race			
Caucasian	50 (76.9%)	11 (61.1%)	78 (71.6%)
Indigenous/Polynesian	2 (3.1%)	0	8 (7.3%)
Asian/Indian	13 (20.0%)	6 (33.3%)	21 (19.3%)
Other	1 (1.5%)	1 (5.6%)	2 (1.8%)
Cause of end stage kidney disease			
Diabetic or renovascular	11 (16.7%)	1 (5.6%)	8 (7.3%)
Polycystic kidney disease	9 (13.6%)	5 (27.8%)	15 (13.8%)
Glomerulonephritis	35 (53.0%)	6 (33.3%)	58 (53.2%)
Other	11 (16.7%)	6 (33.3%)	28 (25.7%)
Re-transplant	7 (10.8%)	0	7 (6.4%)
Preemptive transplant	20 (31.2%)	7 (38.9%)	38 (34.9%)
Peak PRA >80%	3 (7.5%)	0	0
Pre-transplant DSA	28 (42.4%)	4 (22.2%)	34 (31.2%)
MFI of immunodominant DSA			
≥2000	4 (6.1%)	0	11 (10.1%)
<2000	24 (36.4%)	4 (22.2%)	23 (21.1%)
Blood group antibody titer pre-treatment (median, IQR)	16.0 (5.0–56.0)	16.0 (4.0–32.0)	-
Blood group antibody titer on day of transplant (median, IQR)	1.0 (1.0–2.0)	1.5 (0.0–2.0)	-
Male donor	21 (32.3%)	5 (27.8%)	46 (42.6%)
Donor age in years (mean, SD)	52.2 (11.8)	46.0 (8.6)*	48.6 (11.3)*
HLA A/B/DR mismatch (mean, SD)	3.8 (1.6)	3.5 (1.5)	3.0 (1.8)*
Delayed graft function	3 (4.5%)	1 (5.6%)	2 (1.8%)
Ischemic time in hours (mean, SD)	3.9 (1.3)	4.2 (0.9)	4.2 (1.3)
Induction			
Basiliximab	66 (100%)	18 (100%)	105 (96.3%)
Thymoglobulin	0	0	2 (1.8%)
Triple immunosuppression	66 (100%)	18 (100%)	104 (95.4%)
Desensitization	66 (100%)	18 (100%)	18 (16.5%)
Rituximab	0	18 (100%)	0
Intravenous immunoglobulin	66 (100%)	18 (100.0%)	17 (15.6%)
Plasma exchange	7 (10.6%)	1 (5.6%)	6 (5.5%)
Column immunoadsorption	51 (77.3%)	14 (77.8%)	0

*p < 0.05 for comparison to rituximab-free ABOiKT group; all other comparisons to rituximab-free ABOiKT are non-significant.

### Immunological Characteristics

All combinations of blood group incompatibility were represented in the ABOiKT cohort except AB to O. Thirty-nine (59%) rituximab-free ABOiKT recipients were transplanted against anti-A antibodies compared to 13 (72%) for ABOiKT + R. The median baseline anti-blood group antibody titer was 1:16 (range 1:1–1:512) in the rituximab-free ABOiKT group and 1:16 (range 1:1–1:256) in ABOiKT + R. The median number of immunoadsorption sessions required pre-transplant was 3 (range 0–8). All ABOiKT recipients achieved a titer of 1:8 or less at the time of transplant.

The mean number of HLA A, B and DR mismatches were 3.8 (SD 1.6) amongst rituximab-free ABOiKT compared to 3.5 (SD 1.5) for ABOiKT + R and 3.0 (SD 1.8) for ABOcKT. Regarding anti-HLA antibodies, only three recipients, all in the rituximab-free ABOiKT group, had a calculated panel reactive antibody (cPRA) greater than 80% (range 85%–96%). The prevalence of pre-transplant donor specific anti-HLA antibodies was 42% in rituximab-free ABOiKT, 22% in ABOiKT + R and 31% in ABOcKT. The large majority of pre-transplant DSAs were weak with MFI<2000, further details on DSA characteristics are included in Supplementary Table S2.

### Rejection

Over the whole follow-up period, treated biopsy-proven rejection occurred in 30 (46%) rituximab-free ABOiKT, 4 (22%) ABOiKT + R and 28 (26%) ABOcKT recipients. Early rejection, defined as any treated episode of acute rejection within 3 months of transplant, occurred in 26 (39%) rituximab-free ABOiKT, 1 (6%) ABOiKT + R and 16 (15%) ABOcKT recipients. The histological type of the first rejection episode and Banff classifications are shown in Table 2.

**TABLE 2:** Characteristics of first acute rejection episodes.

	ABOiKT		*p*-value^a^	ABOcKT *n* = 109	*p*-value^b^
	Rituximab-free *n* = 66	Rituximab *n* = 18			
Any acute rejection	30 (45.5%)	4 (22.2%)	0.11	28 (26%)	0.001
Time to first rejection, days (median, IQR)	8 (6–47)	1,048 (568–1,387)	0.04	77 (10–375)	0.01
T-cell mediated rejection	24 (36%)	4 (22.2%)	0.40	25 (23%)	0.06
Banff Score					
Borderline	12 (18.2%)	1 (5.6%)	0.66	7 (6.4%)	0.27
IA	5 (7.6%)	2 (11.1%)		11 (10.1%)	
IB	2 (3.0%)	0 (0%)		1 (0.9%)	
IIA	5 (7.6%)	1 (5.6%)		6 (5.5%)	
Antibody mediated rejection	11 (16.7%)	0	0.11	8 (7.3%)	0.08

^a^ ABOiKT with rituximab compared to rituximab-free ABOiKT.

^b^ ABOcKT compared to rituximab-free ABOiKT.

No episodes of antibody mediated rejection (AMR) were observed in the ABOiKT + R cohort compared to 11 (17%) in rituximab-free ABOiKT and 8 (7%) in ABOcKT. Six rituximab-free ABOiKT recipients experienced AMR refractory to maximal medical therapy and required splenectomy at a mean of 22 days post-transplant (range 9–55 days). All but one of these recipients had a rebound of anti-blood group antibody titer to greater than 1:8 coinciding with the diagnosis of rejection. All achieved eventual resolution of AMR without acute graft loss at last follow-up. No comparable episodes of refractory AMR occurred in the ABOcKT cohort.

Results of the univariable analysis of factors associated with rejection are shown in Supplementary Table S1. Results of the multivariable analysis of all rejection over the follow-up period and early rejection are shown in Table 3. When controlling for sex, HLA mismatch, pre-transplant DSA and donor and recipient age, there was no significant difference in acute rejection over the whole follow-up period between rituximab-free ABOiKT and ABOiKT + R (OR 2.5, 95% CI 0.7–8.7, *p* = 0.2). There was a trend toward increased risk of rejection in rituximab-free ABOiKT compared to ABOcKT (OR 2.0, 95% CI 1.0–3.9, *p* = 0.06). Older donor age (OR 1.5 for every 10 years increment in age, 95% CI 1.1–2.1, *p* = 0.02) and HLA mismatch (OR 1.3 for each additional HLA-ABDR mismatch, 95% CI 1.0–1.6, *p* = 0.04) were independent risk factors for rejection in this analysis.

**TABLE 3:** Multivariable logistic regression models of treated acute rejection episodes over the whole follow-up period and early acute rejection (within 3 months of transplant).

	Odds ratio	95% Confidence interval	*p*-value
Any acute rejection episode			
Rituximab-free ABOiKT vs. ABOcKT	2.0	1.0–3.9	0.06
Rituximab-free ABOiKT vs. ABOiKT with rituximab	2.5	0.7–8.7	0.2
Age at transplantation (per 10 years)	0.8	0.6–1.0	0.07
Sex (male)	1.8	0.9–3.9	0.1
Donor age (per 10 years)	1.5	1.1–2.1	0.02
Total mismatch at HLA A, B, DR	1.3	1.0–1.6	0.04
Pre-transplant DSA	1.1	0.6–2.3	0.7
Early rejection			
Rituximab-free ABOiKT vs. ABOcKT	2.9	1.4–6.2	0.005
Rituximab-free ABOiKT vs. ABOiKT with rituximab	8.8	1.1–73.1	0.04
Total mismatch at HLA A, B, DR	1.4	1.1–1.8	0.006
Donor age (per 10 years)	1.3	0.9–1.8	0.2
Pre-transplant DSA	1.3	0.6–2.8	0.5

Early rejection occurred in significantly more rituximab-free ABOiKT recipients compared to both ABOiKT + R (OR 8.8, 95% CI 1.1–73.1, *p* = 0.04) and ABOcKT (OR 2.9, 95% CI 1.4–6.2, *p* = 0.005), controlling for donor age, HLA mismatch and pre-transplant DSA.

Rebound of Anti-A or B antibody titer >1:8 post-transplant occurred in 13 ABOiKT recipients (16%) after a median of 7 days (IQR 3–9, range 1–15) and was strongly associated with incidence of rejection (OR 6.5, 95% CI 1.8–31.2, *p* = 0.008, see also Supplementary Table S1). None of the patients who received pre-transplant rituximab experienced an antibody rebound >1:8.

The presence of a pre-transplant DSA was not significantly associated with all rejection or early rejection on univariable analysis (*p* = 0.26 and 0.15 respectively). Supplementary Table S3 shows associations between various pre-transplant DSA characteristics and rejection, none of which are statistically significant. Detection of a *de novo* DSA was significantly more common in those recipients who experienced rejection compared to those who did not (39%, *n* = 22, compared to 14%, *n* = 15, univariable *p* < 0.001).

### Transplant Outcome

Two recipients, both rituximab-free ABOiKT, experienced early graft loss. A 58 year-old man with immediate graft function incurred graft loss at day six, despite treatment with methylprednisolone and immunoadsorption, caused by severe AMR (proven histologically post-nephrectomy) associated with anti-A rebound. Secondly, a 34 year-old man experienced delayed graft function then developed unexplained fevers before loss of graft perfusion was noted on ultrasound on post-operative day five. Histological examination of the graft was inconclusive as to the presence of rejection due to extensive necrosis.

Death censored graft survival (DCGS) at 1 year was 95% (95% CI 89%–100%) for the rituximab-free ABOiKT group compared to 100% in both the ABOiKT + R and ABOcKT groups. DCGS at 3 years was 90% (95% CI 80%–99%) in rituximab-free ABOiKT compared to 100% and 95% (95% CI 90%–99%) in ABOiKT + R and ABOcKT respectively, with no significant differences between groups. DCGS was strongly associated with prior rejection (HR 4.5, 95% CI 1.38–14.5, *p* = 0.013).

Patient survival was not different between groups. There were two deaths with a functioning graft in rituximab-free ABOiKT, at 1,316 days from an unknown cause and 2,105 days from post-transplant lymphoproliferative disorder; two deaths after ABOiKT + R, at 61 days from infection and 1,558 days from suicide; and one death with functioning graft 415 days after ABOcKT from infection. Three year overall patient survival was 95% (95% CI: 88%–100%) in rituximab-free ABOiKT, 94% (95% CI: 84%–100%) in ABOiKT + R and 99% (95% CI: 97%–100%) in ABOcKT.

### Infection

Data on incidence of infection requiring hospitalization was available for ABOiKT recipients only. There were 74 episodes of infection requiring hospitalization in 31 ABOiKT recipients. Fifty (68%) of these were bacterial infections, 13 (18%) viral and 6 (8%) fungal with the remainder having no organism isolated. Those who experienced treated acute rejection were more likely to have an infection requiring hospitalization (OR 2.6, 95% CI 1.0–6.5, *p* = 0.04), while receipt of rituximab was not associated with infection.

## Discussion

In this single-center, retrospective cohort study, ABOiKT performed without rituximab in the desensitization protocol were more likely to experience early rejection than recipients of either an ABOiKT performed with rituximab or an ABOcKT. Prominent among early rejections were six episodes of severe AMR requiring salvage splenectomy and at least one causing graft failure at day 6, all in the rituximab-free ABOiKT group. The association between rituximab-free ABOiKT and early rejection remained significant when controlling for baseline risk factors including donor age, degree of HLA mismatch and presence of a DSA pre-transplant. Patient and graft survival were not different between groups; however, this study was underpowered to detect such differences at the median follow-up of 3.1 years.

The putative benefit of B-cell depletion in ABOiKT protocols is to reduce the risk of post-transplant rebound of graft-threatening blood group antibodies [20, 21]. In support of this, we observed blood group antibody rebound only in the rituximab-free ABOiKT group and rebound was strongly associated with incidence of rejection.

There are no randomized trials examining the benefit of B-cell depleting therapy in ABOiKT thus the evidence base is reliant on observational studies. A large international registry study of ABOiKT in which splenectomy was very rare (*n* = 11), found that 3 years DCGS was significantly better in the 1,058 patients who received anti-CD20 therapy compared to the 125 who did not [22]. Conversely, in a smaller 2009 study, Montgomery et al. [23] reported equivalent outcomes for 28 patients who underwent ABOiKT with no B-cell depleting therapy at Johns Hopkins Hospital compared to 32 ABOiKT from an earlier era where rituximab or splenectomy were in use. The hitherto largest published cohort of ABOiKT performed without splenectomy or rituximab (*n* = 54) was from The Royal Melbourne Hospital, Australia [13, 24]. At 1 year follow-up they reported rejection in 19% of rituximab-free ABOiKT which was comparable to contemporaneous ABOcKT (17%) and there were no episodes of refractory AMR or graft loss. This group also published a case series of successful ABOiKT (*n* = 20) performed with neither B-cell depleting therapy nor extracorporeal antibody removal in selected recipients with low baseline blood group antibody titers [25]. Recipients with preformed HLA DSA were excluded from both cohorts, in contrast to our practice, thus it is possible that lower HLA immune risk was an important factor in their reported success with rituximab-free ABOiKT.

Excess infection risk conferred by the ABOiKT desensitization protocol remains a concern [26, 27]. Increased infection related deaths have been reported in ABOiKT compared to ABOcKT recipients in a meta-analysis of observational studies and a large multi-national registry both examining the post-splenectomy era [9, 22]. Increased rates of serious infection have been observed in standard compared to low dose rituximab in ABOiKT [28, 29] and in ABOcKT treated with rituximab for various indications [30, 31]. We did not observe an excess of infections requiring hospitalization in ABOiKT who received rituximab compared to those who did not, although the numbers available for comparison in the rituximab group are small. The factor with the strongest association with infection was incidence of rejection, which likely reflects the downstream effects of increased immunosuppression used to treat this complication.

The decision to undertake kidney transplantation across the blood group barrier ultimately depends on the timely availability of alternative transplant options. Prospective ABOiKT recipients in Australia have the option to seek an ABOc transplant through either the national paired kidney exchange program or waitlisting for a disease donor organ. Both alternatives can entail significant additional waiting time with attendant risk of morbidity and mortality due to complications of ESKD [3]. Thus, our center’s ABOiKT program remains active, however, rituximab was reintroduced into the conditioning protocol from August 2019 after review of the outcomes reported herein.

The limitations of this study include the retrospective observational design and the small number in the ABOiKT + R cohort. Although immunosuppression practices did not change apart from the exclusion of rituximab, there is residual risk of confounding by era when comparing the two ABOiKT cohorts. For instance, numerically more DSA-positive ABOiKT recipients were present in the later rituximab-free cohort, which may reflect both a greater leniency in candidate selection over time and the limited donor pool for sensitized individuals in our system. The overall prevalence of DSAs in this cohort may limit the generalizability of our results. For instance, it is possible that the benefit of rituximab seen here was also due to mitigation of HLA-associated immune risk rather than solely that due to ABO incompatibility. Notably, pre-transplant DSA was not associated with rejection in this cohort, likely in part because recipients with moderate to high level antibodies were either directed toward alternate donors or offered enhanced immunosuppression. Nonetheless, rituximab-free ABOiKT remained significantly associated with early rejection while controlling for HLA mismatch and DSA positivity in the multivariable analysis. Moreover, we repeated our analyses on the subgroup with no preformed DSAs and there remained significantly more early rejection in rituximab free ABOiKT compared to ABOiKT + R and ABOcKT (Supplementary Table S4). Finally, the protocolization of an allograft biopsy at day 10 in ABOiKT but not ABOcKT recipients raises the possibility of increased detection of subclinical rejection in the former. On the contrary, review of patient records indicates that there was clinical suspicion of rejection motivating 18 out of the 20 biopsies diagnosing rejection in ABOiKT within 2 weeks of transplant.

In conclusion, we report the largest published single-center cohort of ABOiKT performed without B-cell depleting therapy. Rituximab-free ABOiKT recipients experienced significantly more early acute rejection than ABOiKT performed with rituximab and ABOcKT. Ideally, a randomized controlled trial would be performed to assess the safety and utility of rituximab for ABOiKT. In the absence of such a study, best practice will rely on observational data and on this basis our findings support the inclusion of rituximab for ABOiKT desensitization protocols.

## Data Availability

The raw data supporting the conclusion of this article will be made available by the authors, without undue reservation.

## Abbreviations

ABOcKT, ABO compatible kidney transplant; ABOiKT, ABO incompatible kidney transplant; ABOiKT + R, ABO incompatible kidney transplant with rituximab; AMR, antibody mediated rejection; CDC, complement dependent cytotoxic; CI, confidence interval; DGF, delayed graft function; DSA, donor specific antibody; ESKD, end stage kidney disease; HLA, human leukocyte antigen; HR, hazard ratio; IQR, interquartile range; IV, intravenous; IVIg, intravenous immunoglobulin; OR, odds ratio; cPRA, calculated panel reactive antibody; SD, standard deviation.
