# Supplementary material for: ABO Incompatible Kidney Transplantation Without B-cell Depletion is Associated With Increased Early Acute Rejection: A Single-Center Australian Experience
Source: Transpl Int. 2023 Sep 20;36:11567. doi: 10.3389/ti.2023.11567 (PMC10547868; doi:10.3389/ti.2023.11567)
Supplement: Supplementary file 1 [file DataSheet1.docx]

Supplementary appendix

Table S1: Univariable analysis of factors associated with rejection over the whole follow-up period

|  |  | Rejection | |  |
| --- | --- | --- | --- | --- |
| Variable |  | No | Yes | p-value |
| Transplant cohort | Rituximab-free ABOiKT  ABOiKT+R  ABOcKT | 36 (27.5)  14 (10.7)  81 (61.8) | 30 (48.4)  4 (6.5)  28 (45.2) | 0.02 |
| Age at transplant (years) | Mean (SD) | 46.2 (13.3) | 45.9 (16.4) | 0.907 |
| Sex | Male | 85 (64.9) | 48 (77.4) | 0.096 |
| Cause of ESKD | Diabetic nephropathy | 6 (4.6) | 8 (12.9) | 0.290 |
|  | Renovascular disease | 3 (2.3) | 3 (4.8) |  |
|  | Reflux nephropathy | 9 (6.9) | 6 (9.7) |  |
|  | Polycystic kidney disease | 19 (14.5) | 10 (16.1) |  |
|  | Glomerulonephritis | 73 (55.7) | 26 (41.9) |  |
|  | Unknown cause | 6 (4.6) | 3 (4.8) |  |
|  | Other | 15 (11.5) | 6 (9.7) |  |
| Race | Caucasian | 93 (71.5) | 46 (74.2) | 0.836 |
|  | ATSI/Polynesian | 6 (4.6) | 4 (6.5) |  |
|  | Asian/Indian | 29 (22.3) | 11 (17.7) |  |
|  | Other | 2 (1.5) | 1 (1.6) |  |
| Donor age (years) | Mean (SD) | 48.2 (11.8) | 52.5 (10.1) | 0.01 |
| Total mismatches at HLA- A, B and DR | Mean (SD) | 3.2 (1.8) | 3.8 (1.5) | 0.02 |
| Peak PRA (%) | Median (IQR) | 1.0 (0.0 to 6.0) | 3.0 (0.0 to 7.0) | 0.188 |
| Pre-formed DSA | Yes | 41 (31.8) | 25 (40.3) | 0.256 |
| De novo DSA | Yes | 15 (13.8) | 22 (39.3) | <0.001 |
| Duration on dialysis pre-transplant (years) | Mean (SD) | 1.6 (2.4) | 2.5 (6.1) | 0.272 |
| Delayed graft function | Yes | 4 (3.1) | 2 (3.2) | 1.000 |
| Rebound of blood group antibodies >1:8 (ABOi only) | Yes | 3 (6.0) | 10 (29.4) | 0.009 |
| Max blood group antibody titer post-transplant (ABOi only) | Median (IQR) | 4.0 (1.0 to 8.0) | 8.0 (2.5 to 16.0) | 0.011 |

Table S2 Characteristics of pre-transplant DSAs in the three treatment groups. There were no significant differences in any of these variables when comparing rituximab-free ABOiKT to ABOiKT+R or ABOcKT (P>0.05 for all comparisons). * These patients did not have a DSA on serum tested within one month of transplant but were positive for one or more DSAs on historical sera.

|  |  | Rituximab-free ABOiKT  n=28 | ABOiKT+R  n=4 | ABOcKT  n=34 |
| --- | --- | --- | --- | --- |
| Historical DSA only* | Yes | 5 (17.9) | 0 (0.0) | 9 (26.5) |
| Pre-transplant DSA class | Class I | 12 (42.9) | 2 (50.0) | 10 (29.4) |
|  | Class II | 11 (39.3) | 1 (25.0) | 15 (44.1) |
|  | Class I&II | 5 (17.9) | 1 (25.0) | 9 (26.5) |
| Number of DSA specificities | Median (IQR) | 1.0 (1.0 to 2.0) | 1.0 (1.0 to 1.2) | 1.5 (1.0 to 3.0) |
| MFI of immunodominant DSA | Median (IQR) | 1016.0 (681.2 to 1559.2) | 817.0 (563.2 to 1203.2) | 1279.5 (717.2 to 2322.0) |
| Immunodominant DSA MFI category | MFI ≥ 3000 | 1 (3.6) | 0 (0.0) | 4 (11.8) |
|  | MFI 1500-3000 | 6 (21.4) | 1 (25.0) | 9 (26.5) |
|  | MFI 500-1500 | 21 (75.0) | 3 (75.0) | 21 (61.8) |

Table S3 Association of pre-transplant DSA characteristics with rejection over the whole follow-up period. Additionally, none of these variables were significant when the outcome was early rejection or antibody mediated rejection (data not shown).

|  |  | **Rejection** | |  |
| --- | --- | --- | --- | --- |
|  |  | No | Yes | p |
| **All cases:** |  |  |  |  |
| Pre-transplant DSA | Yes | 41 (31.3) | 25 (40.3) | 0.256 |
| MFI of immunodominant DSA | Median (IQR) | 0.0 (0.0 to 637.5) | 0.0 (0.0 to 738.2) | 0.191 |
| MFI of immunodominant DSA (categorical) | MFI≥2000 | 8 (6.1) | 7 (11.3) | 0.333 |
|  | MFI<2000 | 33 (25.2) | 18 (29.0) |  |
|  | Negative | 90 (68.7) | 37 (59.7) |  |
| **DSA positive cases only:** |  |  |  |  |
| MFI of immunodominant DSA | Median (IQR) | 1139.0 (697.5 to 1682.5) | 821.0 (675.5 to 2080.5) | 1.000 |
| Pre-transplant DSA class | Class I | 16 (39.0) | 8 (32.0) | 0.686 |
|  | Class II | 15 (36.6) | 12 (48.0) |  |
|  | Class I & II | 10 (24.4) | 5 (20.0) |  |

Table S4 Sensitivity analysis with pre-transplant DSA cases excluded. Characteristics of first acute rejection episodes. * ABOiKT with rituximab compared to rituximab- free ABOiKT; ** ABOcKT compared to rituximab-free ABOiKT.

|  | ABOiKT | | p-value* | ABOcKT | p-value** |
| --- | --- | --- | --- | --- | --- |
|  | Rituximab-free  n=38 | Rituximab  n =14 |  | n=75 |  |
| *Any acute rejection* | 17 (44.7%) | 4 (29%) | 0.35 | 16 (21%) | 0.02 |
| *Early Rejection within 3 months* | 14 (36.8%) | 1 (7.1%) | 0.04 | 9 (12%) | 0.003 |
| *Time to first rejection, days (median, IQR)* | 9 (7-74) | 1048 (568-1387) | 0.08 | 78 (10-505) | 0.14 |
| *T-cell mediated rejection* | 14 (37%) | 4 (29%) | 0.74 | 15 (20%) | 0.07 |
| *Banff Score* |  |  |  |  |  |
| *Borderline* | 6 | 1 | 0.66 | 4 | 0.6 |
| *IA* | 3 | 2 |  | 5 |  |
| *IB* | 2 | 0 |  | 1 |  |
| *IIA* | 3 | 1 |  | 5 |  |
| *Antibody mediated rejection* | 7 (18.8%) | 0 | 0.17 | 5 (6.7%) | 0.1 |
